# Hospitalizations for diarrhea in children under 5 years of age: time and spatial series analysis, Arapiraca, 2010–2021

**DOI:** 10.1590/S2237-96222026v35e20250725.en

**Published:** 2026-07-24

**Authors:** Evandro da Silva Melo, Carla Souza dos Anjos, Lousanny Caires Rocha Melo, Rafaella Souza Albuquerque

**Affiliations:** 1 Universidade Federal Fluminense Faculdade de Medicina Niterói RJ Brazil Universidade Federal Fluminense, Faculdade de Medicina, Niterói, RJ, Brazil; 2 Universidade Federal de Alagoas Programa de Pós-Graduação em Enfermagem Maceió AL Brazil Universidade Federal de Alagoas, Programa de Pós-Graduação em Enfermagem, Maceió, AL, Brazil

**Keywords:** Diarrhea, Gastroenteritis, Epidemiology, Time Series Studies, Spatial Analysis, Diarrea, Gastroenteritis, Epidemiología, Estudios de Series Temporales, Análisis Espacial

## Abstract

**Objective:**

To analyze the temporal trend and spatial distribution of hospitalizations for diarrhea in children under 5 years of age in Arapiraca, Alagoas, between 2010 and 2021.

**Methods:**

This is a time and spatial series analysis of hospitalizations for diarrhea in children under 5 years of age in Arapiraca. The temporal trend in hospitalization rates was assessed using autoregressive linear regression from 2010 to 2019, estimating the average annual percent change (AAPC), the 95% confidence interval (95%CI), and the corresponding p-value. A density map was created to assess spatial distribution using the Kernel density estimator, identifying case clusters between 2017 and 2021.

**Results:**

A total of 4,013 hospitalizations were recorded; 59.9% occurred in children under 2 years of age, 57.1% remained hospitalized for 2 to 3 days, and there were 7 deaths. In 2021, hospitalization rates in children under 1 year of age were 4 times higher in the rural area (40.3/1,000 children/year; 95%CI 26.8; 64.7). The trend was increasing for hospitalizations in children under 5 years of age in rural (AAPC 0.56; 95%CI 0.29; 0.83; p-value 0.001) and urban areas (AAPC 0.06; 95%CI 0.02; 0.09; p-value 0.006), and in children aged 1 to 4 years, remaining stationary among those under 1 year of age. Spatial analysis identified clusters of cases in areas of accelerated urbanization.

**Conclusion:**

Diarrhea is one of the main causes of hospitalization in childhood, with increasing trends in vulnerable territories, reinforcing the need for health surveillance and planning.

## Resumen

**Objetivo:**

Analizar la tendencia temporal y la distribución espacial de las hospitalizaciones por diarrea en niños menores de 5 años en Arapiraca, Alagoas, entre 2010 y 2021.

**Métodos:**

Se trata de un análisis de series temporales y espaciales de hospitalizaciones por diarrea en menores de 5 años en Arapiraca. La tendencia temporal de las tasas de hospitalización fue evaluada mediante regresión lineal autorregresiva entre 2010 y 2019, estimándose la variación porcentual anual promedio (VPAP), el intervalo de confianza del 95% (IC95%) y el respectivo valor de p. Se construyó un mapa de densidad para evaluar la distribución espacial utilizando el estimador de densidad de Kernel, con el fin de identificar conglomerados de casos entre 2017 y 2021.

**Resultados:**

Se registraron 4.013 hospitalizaciones; el 59,9% fueron en niños menores de 2 años, el 57,1% permanecieron hospitalizados entre 2 y 3 días y hubo 7 defunciones. En 2021, las tasas de hospitalización en menores de 1 año fueron 4 veces superiores en la zona rural (40,3/1 mil niños/año; IC95% 26,8; 64,7). La tendencia fue creciente en las hospitalizaciones de niños menores de 5 años en las zonas rurales (VPAP 0,56; IC95% 0,29; 0,83; valor de p 0,001) y en las zonas urbanas (VPAP 0,06; IC95% 0,02; 0,09; valor de p 0,006), y en niños de 1 a 4 años, manteniéndose estacionaria en los menores de 1 año. El análisis espacial identificó conglomerados de casos en áreas de urbanización acelerada.

**Conclusión:**

La diarrea constituye uno de los principales problemas que motivan la hospitalización infantil, con tendencia ascendente en territorios vulnerables, lo que refuerza la necesidad de vigilancia y planificación en salud.

**Ethical aspects**

This research respected ethical principles, having obtained the following approval data:

Research ethics committee Universidade Federal Fluminense

Opinion number 5.753.066

Approval date 10/11/2022

Certificate of submission for ethical appraisal 64248922.0.0000.5243

Informed consent form Exempt.

## Introduction

Diarrhea and gastroenteritis of presumed infectious origin are infectious diseases of the gastrointestinal tract characterized by increased stool frequency and watery stools. In the International Statistical Classification of Diseases and Related Health Problems, 10th Revision (ICD-10), its code is A09 [1].

In 2021, diarrheal diseases were estimated to cause approximately 1.17 million deaths worldwide (95% confidence interval, 95%CI 0.793; 1.62 million), representing a 60% reduction compared to 1990, when this number was estimated at 2.93 million. The most pronounced decrease was observed in children under 5 years of age, whose diarrhea-related mortality declined by approximately 79% [2]. In the Federal District (2003–2012) and nationwide (2000–2015), it was observed that the magnitude of hospitalization rates due to this cause is high among children under 1 year of age [3,4], with a substantial impact on the entire population under 5 years of age [5].

Among the variables associated with diarrhea are the geographic area where the child resides, the number of residents per room, the ages of the mother and child [6], the lack of a street-level sewage system, inadequate solid waste storage, and the presence of intradomiciliary vectors [6,7]. In addition to etiological factors of viral and bacterial origin, family income, attendance at daycare/school [7], and lack of access to safe drinking water are also included [6]. Access to primary health care services is considered a protective factor against diarrhea [7]. Vaccination against rotavirus is an important protective measure, associated with a significant reduction in hospitalizations and deaths from diarrhea among children under 5 years of age in Brazil [8].

In Mato Grosso, between 2011 and 2020, geospatial clustering of hospitalizations for diarrhea among children was observed, indicating municipal heterogeneity and suggesting that local factors (sanitary, socioeconomic, and access to health care) may modulate the risk of hospitalization [9]. In Arapiraca, between 2012 and 2021, 4,839 hospitalizations of children under 5 years of age due to primary care-sensitive conditions were recorded. Of these, 75% occurred due to diarrhea [10].

Despite advances in controlling diarrheal diseases, understanding territorial inequalities in pediatric hospitalizations remains relevant for planning surveillance and prevention efforts. This study aimed to analyze the temporal trend and spatial distribution of hospitalizations for diarrhea in children under 5 years of age in Arapiraca, Alagoas, between 2010 and 2021.

## Methods

### Study design

This study uses time series and spatial analyses of hospitalizations for diarrhea (ICD-10 A09) among children under 5 years of age residing in and hospitalized in Arapiraca, Alagoas. The temporal trend analysis considered the period 2010–2019, based on rates aggregated by age group and area of residence. The spatial analysis included hospitalizations from 2017 to 2021 and used georeferenced and anonymized addresses to describe the spatial distribution of events. 

### Setting

The study was conducted in Arapiraca, a municipality in the agreste region of Alagoas. In 2022, the Brazilian Institute of Geography and Statistics (*Instituto Brasileiro de Geografia e Estatística*, IBGE) estimated a population of 234,696 inhabitants, of whom 7.2% were children under 5 years of age. The infant mortality rate was 14.05 deaths per 1,000 live births in 2021 and 14.19 in 2023. 

Regarding household conditions, 20.7% of households had access to a sewage system, 77.6% were supplied by the public water system, and 98.6% had a bathroom for exclusive household use [11]. Between 2012 and 2021, 75.0% of hospitalizations for primary care-sensitive conditions in children under 5 years of age were attributed to diarrhea, according to the National Hospital Information System of the Brazilian Unified Health System (*Sistema de Informações Hospitalares do Sistema Único de Saúde*, SIH/SUS) [10].

### Participants

All hospitalizations recorded in the SIH/SUS for children under 5 years of age residing in Arapiraca, Alagoas, with an underlying cause of ICD-10 A09 were included, considering only primary diagnoses. This option increases specificity, although it may underestimate the number of cases classified as secondary diagnoses. 

Hospitalizations performed in public, private, or philanthropic facilities that provide services to the Brazilian Unified Health System (*Sistema Único de Saúde*, SUS) in the municipality were included. The criterion of analyzing only residents hospitalized within the municipality was adopted because the municipal SIH database provided complete geographic variables. 

During the analyzed period, 4,020 hospitalizations were recorded, of which 4,013 (99.8%) occurred in hospitals in Arapiraca. Each hospitalization was treated as an independent event, without identifying possible readmissions. This may overestimate the number of individual hospitalizations, but it reflects the pattern of health service utilization. All records contained valid age information.

### Variables, data sources, and measurement

The information used in the descriptive and temporal trend analyses was obtained from the SIH/SUS, accessed through the portal of the Brazilian Unified Health System Information Technology Department (*Departamento de Informática do Sistema Único de Saúde*, DATASUS) [11].

The variables analyzed included:

- hospitalization facility (Casa de Saúde e Maternidade Nossa Senhora de Fátima, Hospital Regional de Arapiraca, Hospital Chama, Real Hospital Santa Maria);
- age group (0–<1, 1–<2, 2–<3, 3–<4, 4–<5 years);
- sex (male, female);
- race/skin color (White, Black, Brown [Brazilian mixed race], Asian, Indigenous, unknown);
- discharge status (discharge, continued stay due to disease characteristics, transferred to another facility, death, administrative case closure); and
- length of hospital stay (≤1, 2–3, 4–5, ≥6 days).

The analyses were stratified according to area of residence (rural, urban, unknown). The race/skin color variable had missing information in 13.1% of the records; the “unknown” category was retained in the descriptive tables.

For the spatial analysis, the local municipal SIH database was used, which contains additional information not available in DATASUS, such as postal code and street address. Therefore, it was possible to geocode the cases and produce maps. The data were extracted in March 2023 and analyzed in anonymized form. 

For the calculation of rates, the numerator corresponded to hospitalizations with a primary diagnosis of ICD-10 A09 recorded in the SIH/SUS between 2010 and 2021. The denominator was the annual estimated population of children under 5 years of age, stratified by age group (<1; 1–4; <5) and area of residence. Rates were expressed per 1,000 children/year.

### Statistical analysis

Pearson’s chi-square test was used to assess associations between the child’s area of residence (rural or urban) and the descriptive variables examined, at a 5% significance level.

To estimate the 95%CI for hospitalization rates, the Poisson distribution method was used, which is treated as a special case of the binomial distribution. This approach was applied when the number of events in the numerator was very small relative to the reference population in the denominator. 

Hospitalization rates were tabulated in a Microsoft Excel spreadsheet after installing the “conf int” add-in, available for download at statpages.info. To calculate the lower confidence interval, the PoisLow formula was used; for the upper confidence interval, the PoisHigh formula was used.

### Estimation of the child population according to rural and urban areas

Given that the IBGE does not provide intercensal estimates stratified by area of residence, this study adopted a methodology for interpolating and extrapolating annual population estimates, assuming constant growth between 2000 and 2021.

The estimated child population for each year (2011–2021) was calculated using the geometric method, in which the population grows proportionally at a constant rate (coefficient r). The reference values for the calculations were data from the 2000 Population Census (P0) and the 2010 Population Census (Pt) [10]. Equation 1 was used to obtain the coefficient r:

Equation 1: $r=[Pt/P0{]}^{\left[1/(t-t0)\right]}-1$

where t corresponded to the number of years counted from 1 August 2010 to 1 August 2021. Equation 2 was used to obtain the projected population: 

Equation 2: ​ $Pt=P0[(1+r{)}^{(t),}]$

where t is the time point to be projected. 

Estimates were obtained for the child population, disaggregated by age group (<1, 1–4, <5) and area of residence (rural, urban). The projected values were compared with the official intercensal estimates from IBGE and showed an average difference of approximately 5%. This discrepancy reflected small local demographic fluctuations not captured by the average intercensal growth, but did not compromise the consistency of temporal trends, since the same method was uniformly applied throughout the analyzed period.

### Temporal trend analysis

This stage began with the inspection of line graphs, in which observed rates were plotted on the y-axis and the years of the series on the x-axis. The analysis of temporal trends in hospitalization rates and autoregressive linear regression were performed using the ordinary least squares method and the “car” and “nlme” packages in R. This method was appropriate for handling the lack of independence of the data (years of the series) and allowed models to be fitted considering temporal lags and adjusting for “n lags” as assessed by the serial autocorrelation function. 

Hospitalization rates for diarrhea were transformed to base-10 logarithms (log₁₀) to stabilize variance and meet the assumption of normality. Two lags (AR(2)) were considered.

The magnitude and direction of the trend were expressed as the average annual percent change (AAPC), calculated from the model’s slope coefficient (β_1_). This was obtained according to the methodological approach adopted by Antunes and Cardoso [12], according to the expression:


$$\mathrm{AAPC}=(10^{\beta_{1}}-1)\times 100$$

The lower and upper limits of the 95%CI were obtained, respectively, by:


LCL=(10β1, lower-1)×100 and UCL=(10β1, upper-1)×100

Trends were classified as increasing when the 95%CI was entirely positive, decreasing when entirely negative, and stationary when it included zero. Temporal trends were analyzed using R software version 4.1.0 (2021-05-18), using data tabulated in Microsoft Excel 2016 spreadsheets. For the temporal trend analysis, the period 2010–2019 was considered.

### Spatial distribution

The spatial distribution of hospitalizations for diarrhea in children under 5 years of age during 2017–2021 was analyzed using the local SIH/SUS database, which provides the postal code and street address of residence. Addresses were geocoded manually using Google Earth Pro (version 7.3; Google LLC), resulting in case-by-case validation of coordinates. The success rate was 90.9%; incomplete or ambiguous addresses were standardized or represented by the street/neighborhood centroid.

The coordinates were converted into a point layer in QGIS 3.16 and overlaid on the municipal census mesh provided by IBGE. Spatial density was estimated using the Kernel method, with a bandwidth of 500 m and the SIRGAS 2000/UTM 24S datum projection. These parameters were selected based on the municipality’s territorial scale to identify areas with higher hospitalization rates.

## Results

Between 2010 and 2021, there were 4,013 hospitalizations for diarrhea in children under 5 years of age in Arapiraca, recorded exclusively in facilities affiliated with the SUS. A single hospital institution accounted for 60.18% (n=2,415) of the hospitalizations (Table 1).

**Table 1:** Quantitative distribution of hospitalizations for diarrhea in children under 5 years of age residing and hospitalized within the scope of the Brazilian Unified Health System (*Sistema Único de Saúde*, SUS), by hospital facility of admission, sex, age group (months), race/skin color, outcome, length of hospital stay (days), and area of residence. Arapiraca, 2010–2021 (n=4,013)

Variables			Area of residence		
		Total n	Unknown n (%)	Rural n (%)	Urban n (%)
**Hospital facility of admission**					
Casa de Saúde e Maternidade Nossa Senhora de Fátima		2,415	34 (51.0)	743 (64.0)	1,638 (59.0)
Hospital Regional de Arapiraca		351	1 (1.5)	81 (7.0)	269 (9.6)
Hospital Chama		867	5 (7.5)	220 (19.0)	642 (23.0)
Real Hospital Santa Maria		380	27 (40.0)	110 (9.5)	243 (8.7)
Sex					
Female		1,943	26 (39.0)	550 (48.0)	1,367 (49.0)
Male		2,070	41 (61.0)	604 (52.0)	1,425 (51.0)
**Age group **(years)					
0–<1		1,221	28 (42.0)	367 (32.0)	826 (30.0)
1–2		1,186	18 (27.0)	351 (30.0)	817 (29.0)
2–<3		755	12 (18.0)	205 (18.0)	538 (19.0)
3–4		516	4 (6.0)	133 (12.0)	379 (14.0)
4–5		335	5 (7.5)	98 (8.5)	232 (8.3)
**Race/skin color**					
White		67	3 (4.5)	20 (1.7)	44 (1.6)
Black		2	-	-	2 (0.1)
Brown (Brazilian mixed race)		3.418	33 (49.3)	992 (86.0)	2,393 (85.7)
Asian		-	-	-	-
Indigenous		-	-	-	-
Unknown		526	31 (46.3)	142 (12.3)	353 (12.6)
Outcome					
Discharge		3,979	67	1,141	2,771
continued stay due to disease characteristics		2	-	1 (<0.1)	1 (<0.1)
Transferred to another facility		24	-	7 (0.6)	17 (0.6)
Death		7	-	5 (0.4)	2 (<0.1)
Administrative case closure		1	-	-	1 (<0.1)
**Length of hospital stay **(days)					
≤1		16	-	5(0.4)	11(0.4)
2–3		2,293	47 (70.0)	691(59.9)	1,555 (55.6)
4–5		1,222	15 (22.0)	332 (28.7)	875 (31.0)
≥6		482	5 (7.5)	126 (10.9)	351 (13.0)

Hospitalizations were more frequent among children under 2 years of age (59.9%), with no significant difference between sexes (p-value 0.457). The length of hospital stay was shorter among children from rural areas. The race/skin color variable showed a predominance of Brown children (85.7%), followed by missing records (13.1%). Incomplete information was more frequent among rural residents (46.3%), highlighting inequalities in data recording quality. There was no statistically significant difference in the distribution of race/skin color between areas of residence (p-value 0.733) (Table 1).

Hospitalization rates were higher in the rural area throughout the entire period, especially among children under 1 year of age, who had the highest incidence rates. In 2021, rates in the rural area were 4 times higher than those in the urban area (40.3 versus 9.9 per 1,000 children/year). Among children under 1 year of age, rates were nearly twice those observed among children aged 1 to 4 years, in both the urban (9.9 vs. 5.0 per 1,000 children/year) and rural (40.3 vs. 21.8 per 1,000 children/year) areas (Table 2).

**Table 2:** Hospitalization rates (HR) for diarrhea (per 1,000 children/year) among children under 5 years of age and their respective 95% confidence intervals (95%CI), within the scope of the Brazilian Unified Health System (*Sistema Único de Saúde*, SUS), stratified by age group, year of hospitalization, and area of residence. Arapiraca, 2010–2021 (n=4,013)

Age group (years)	Year of hospitalization	HR rural area (95%CI)	HR urban area (95%CI)	Total HR (95%CI)
<1	2010	82.0 (60.8; 108.1)	32.1 (25.9; 39.2)	44.1 (37.4; 51.5)
	2011	48.9 (32.8; 70.2)	18.3 (13.7; 24.0)	24.1 (19.2; 29.8)
	2012	46.8 (30.8; 68.1)	21.0 (16.0; 27.1)	26.2 (21.1; 32.3)
	2013	78.6 (57.1; 105.5)	39.8 (32.7; 47.8)	46.8 (39.8; 54.7)
	2014	66.1 (46.3; 91.4)	26.6 (20.9; 33.4)	33.4 (27.5; 40.2)
	2015	54.7 (36.6; 78.6)	22.2 (17.0; 28.6)	28.8 (23.2; 35.2)
	2016	42.7 (26.8; 64.7)	25.8 (20.2; 32.7)	28.9 (23.3; 35.4)
	2017	83.8 (60.4; 113.3)	35.2 (28.4; 43.1)	42.9 (36.0; 50.8)
	2018	73.9 (51.8; 102.3)	28.4 (22.4; 35.7)	36.3 (29.9; 43.6)
	2019	65.5 (44.5; 93.0)	24.2 (18.6; 31.0)	31.0 (25.1; 37.9)
	2020	6.5 (1.3; 19.1)	17.5 (12.8; 23.4)	15.9 (11.7; 21.1)
	2021	40.3 (23.9; 63.6)	9.9 (6.4; 14.6)	14.5 (10.5; 19.5)
1–4	2010	39.6 (31.9; 48.6)	19.0 (16.6; 21.6)	23.1 (20.6; 25.7)
	2011	19.9 (14.5; 26.8)	10.3 (8.6; 12.3)	12.2 (10.4; 14.2)
	2012	26.9 (20.4; 34.9)	14.6 (12.5; 16.9)	16.6 (14.6; 18.9)
	2013	45.7 (36.9; 56.0)	21.1 (18.5; 23.9)	24.9 (22.3; 27.8)
	2014	38.4 (30.2; 48.1)	16.7 (14.4; 19.2)	20.1 (17.7; 22.6)
	2015	22.9 (16.6; 30.9)	10.8 (9.0; 12.9)	12.9 (11.0; 15.0)
	2016	35.5 (27.4; 45.4)	17.5 (15.1; 20.2)	20.5 (18.1; 23.2)
	2017	68.2 (56.5; 81.7)	20.5 (17.9; 23.4)	27.5 (24.7; 30.5)
	2018	42.8 (33.4; 53.9)	16.2 (13.9; 18.7)	20.0 (17.6; 22.7)
	2019	39.5 (30.4; 50.6)	14.9 (12.7; 17.5)	18.5 (16.1; 21.1)
	2020	23.5 (16.5; 32.6)	8.8 (7.1; 10.8)	10.9 (9.1; 12.9)
	2021	21.8 (14.9; 30.7)	5.0 (3.7; 6.5)	7.2 (5.8; 8.9)
<5	2010	48.5 (40.8; 57.2)	21.5 (19.2; 24.0)	27.3 (24.9; 29.8)
	2011	26.1 (20.5; 32.8)	11.9 (10.2; 13.8)	14.6 (12.8; 16.5)
	2012	31.2 (24.9; 38.6)	15.8 (13.9; 18.0)	18.6 (16.6; 20.7)
	2013	52.8 (44.4; 62.5)	24.8 (22.3; 27.5)	29.3 (26.8; 32.0)
	2014	44.5 (36.6; 53.6)	18.6 (16.4; 21.0)	22.8 (20.5; 25.2)
	2015	30.0 (23.4; 37.7)	13.0 (11.2; 15.1)	16.1 (14.2; 18.1)
	2016	37.2 (29.7; 45.9)	19.1 (16.9; 21.6)	22.2 (20.0; 24.6)
	2017	71.8 (61.1; 83.9)	23.4 (20.9; 26.1)	30.6 (27.9; 33.4)
	2018	49.9 (40.9; 60.3)	18.6 (16.3; 21.0)	23.3 (20.9; 25.8)
	2019	45.6 (36.8; 55.8)	16.8 (14.6; 19.1)	21.0 (18.8; 23.4)
	2020	19.6 (14.0; 26.8)	10.5 (8.8; 12.4)	11.9 (10.2; 13.8)
	2021	26.2 (19.4; 34.5)	5.9 (4.7; 7.4)	8.7 (7.2; 10.3)

The temporal behavior was cyclical, with peaks in 2013 and 2017, a decrease in 2020, and an increase in 2021. Rates in the rural area remained consistently higher than those in the urban area, with no overlap of confidence intervals (Figure 1). Analyses from 2010 to 2019 revealed an increasing trend in hospitalization rates for diarrhea among children under 5 years of age, both in the rural area (AAPC 0.56; 95%CI 0.29; 0.83) and in the urban area (AAPC 0.06; 95%CI 0.02; 0.09). Among children aged 1 to 4 years, similar patterns were observed in the rural area (AAPC 0.62; 95%CI 0.38; 0.87) and in the urban area (AAPC 0.05; 95%CI 0.01; 0.09). For children under 1 year of age, the trend remained stationary in the rural area (AAPC 0.19; 95%CI −0.59; 0.99) and increased in the urban area (AAPC 0.06; 95%CI 0.01; 0.11). Considering the overall population of children under 5 years of age, an increasing trend in hospitalizations was observed (AAPC 0.11; 95%CI 0.05; 0.17) (Table 3).

**Table 3:** Temporal trend of hospitalization rates for diarrhea (per 1,000 children/year) among children under 5 years of age and their respective average annual percent changes (AAPC), 95% confidence intervals (95%CI), and p-values, by area of residence and age group. Arapiraca, 2010–2019 (n=3,714)

Area of residence	Age group (years)	Temporal trend	AAPC (95%CI)	p-value
Rural	<5	Increasing	0.56 (0.29; 0.83)	0.001
	<1	Stationary	0.19 (-0.59; 0.99)	0.596
	1–4	Increasing	0.62 (0.38; 0.87)	<0.001
Urban	<5	Increasing	0.06 (0.02; 0.09)	0.006
	<1	Increasing	0.06 (0.01; 0.11)	0.030
	1–4	Increasing	0.05 (0.01; 0.09)	0.014
Total	<5	Increasing	0.11 (0.05; 0.17)	0.004
	<1	Stationary	0.07 (-0.09; 0.23)	0.358
	1–4	Increasing	0.12 (0.07; 0.17)	<0.001

**Figure 1:**
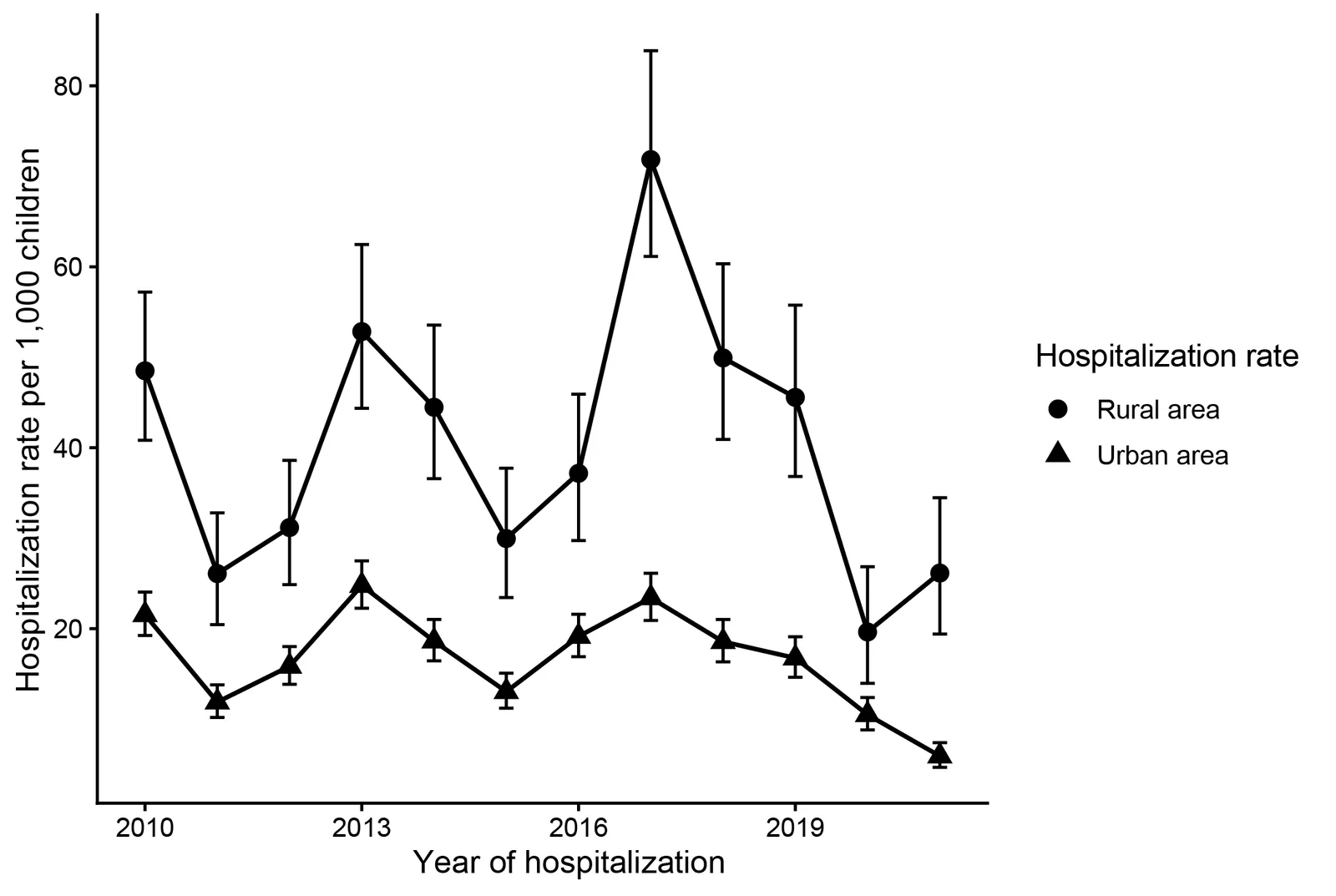
Graphical representation of hospitalization rates for diarrhea (per 1,000 children/year) and their respective 95% confidence intervals (95%CI) among children under 5 years of age, according to year of hospitalization and area of residence. Arapiraca, 2010–2021 (n=4,013)

The spatial analysis of all hospitalizations between 2017 and 2021 identified four high-density clusters, primarily concentrated in the central and southeastern regions of the municipality. Areas of highest density (≥28.5 cases/km^2^, 500 m radius) were located in the Manoel Teles, Primavera, and Olho D’Água dos Cazuzinhas neighborhoods, near Lago da Perucaba, and a fourth cluster was identified in Residencial Agreste, to the north. The first three clusters were located in areas covered by primary health care units, while the fourth was located farther from health services. This finding indicated a spatial mismatch between hospitalization occurrence and health service coverage. This highlighted a non-coincident spatial distribution between hospitalization occurrences and the location of primary health care units (Figure 2).

**Figure 2:**
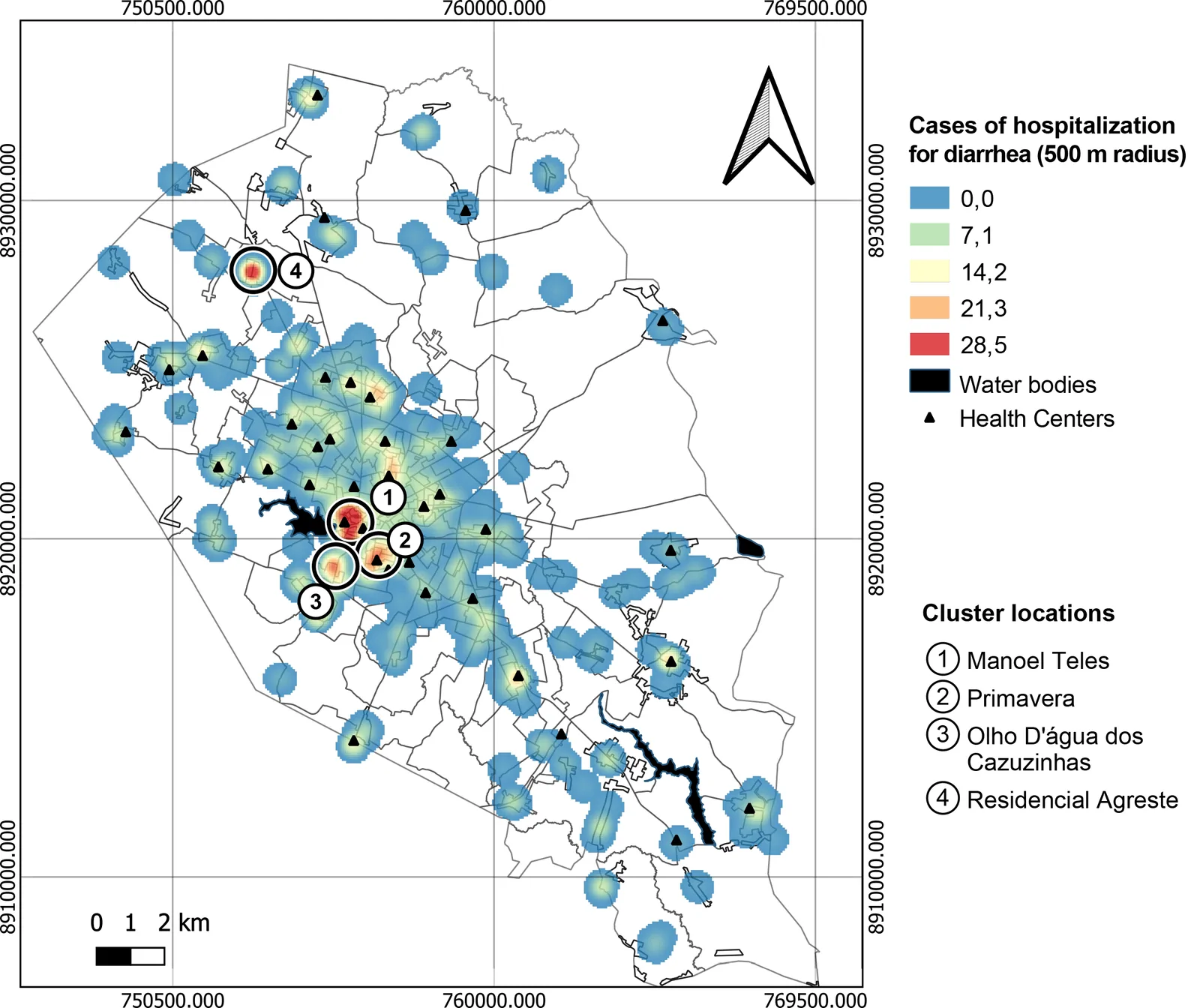
Spatial distribution of hospitalizations for diarrhea among children under 5 years of age. Arapiraca, 2017–2021 (n=1,335)

## Discussion

Hospitalizations for diarrhea in children under 5 years of age in Arapiraca remained high throughout the historical series, with a predominance among children under 2 years of age and a growing trend in rates, especially in rural areas. The identification of clusters of cases in peripheral and recently urbanized areas reinforces the influence of environmental context and household infrastructure on the occurrence of the condition. These findings indicate that diarrhea continues to be an important cause of pediatric hospitalization in the municipality, reflecting territorial and socio-environmental inequalities that influence the risk of illness.

This study presented limitations inherent to the use of secondary data from SIH/SUS, which are subject to underreporting, coding errors, and inconsistencies in the completion of variables, especially address and race/skin color. Possible inaccuracy in the diagnostic coding of ICD-10 A09, which aggregates different causes of diarrhea, may lead to misclassification of cases and affect the accuracy of hospitalization rates. In addition, SIH/SUS includes only hospitalizations financed by the SUS, which may underestimate the occurrence of the condition in exclusively private facilities. 

The population estimates used to calculate rates, based on intercensal projections from IBGE (2000 and 2010 Population Censuses), may also introduce a margin of error. This is particularly true for the years 2020 and 2021, which fall outside the census interval, despite a mean difference of 5% relative to recent official estimates, considered acceptable for municipal-level analyses.

In the spatial analysis, the use of the Kernel density estimator on individual points allowed the identification of spatial clusters of hospitalizations. Still, it did not adjust densities for the population at risk. The concentrations may reflect higher population density of children and/or socio-environmental vulnerability conditions. The results may also have been influenced by the smoothing parameters (bandwidth and cell size) and by geocoding errors arising from the absence or inconsistency of postal codes in some records. 

The high proportion of hospitalizations among children under 2 years of age (60%), with no significant difference between sexes, is consistent with patterns observed in other Brazilian studies. In Ilha de Guaratiba, Rio de Janeiro, in 2016, a similar age pattern was identified, with children under 2.5 years of age at higher risk of diarrhea [7]. Higher hospitalization rates for diarrhea among children under 1 year of age were reported in the Federal District (27%) in 2012 [3]. Although these rates are much lower than those observed in Arapiraca, this finding is particularly relevant considering that acute diarrhea is one of the main causes of childhood morbidity and mortality in Brazil, with a significant impact on quality of life and child development. 

In 2021, it was shown that although the global burden of diarrheal diseases has decreased over the past decades worldwide (a 60% reduction in deaths between 1990 and 2021), significant territorial inequalities persist, concentrated in regions and population groups with poorer infrastructure conditions and lower coverage of health services [2]. In the municipality of São Paulo, epidemiological surveillance of diarrheal diseases identified norovirus as the main etiological agent among laboratory-confirmed cases in the analyzed period. Between 2010 and 2016, 1,565 notifications met the case definition, of which 444 (28%) were confirmed for norovirus and 368 (23%) for rotavirus. These findings reinforce the importance of surveillance of diarrheal diseases, producing information that broadens the understanding of the dynamics of these conditions in childhood [13].

In Arapiraca, the influence of environmental determinants becomes evident when comparing hospitalization rates between rural and urban populations. Access to health services is a key determinant of quality of life and socioeconomic development, and one of the dimensions used by the World Bank in the multidimensional measurement of poverty [14]. Even under similar socioeconomic conditions, access to health services tends to be more limited in rural regions compared to urban areas [15]. In Arapiraca, in 2022, about 92% of the population resided in urban areas; only 21% of households were connected to the sewage system, and 78% had access to water supply through the general network [11]. 

It is noteworthy that this study identified a growing trend in hospitalization rates for diarrhea in children under 5 years of age in Arapiraca, especially in rural areas, contrasting with reduction patterns described in other Brazilian contexts. While in Mato Grosso, annual hospitalization rates for diarrhea among children under 1 year decreased from 8.50 to 3.45 per 1,000 live births between 2011 and 2020 [9], in Arapiraca, the trend remained stationary for the same age group (APC 0.07; 95%CI -0.09; 0.23; p-value 0.358). In Piauí, in the period 2000–2019, a decreasing trend was also observed in hospitalizations and mortality due to acute diarrheal diseases in children under 5 years of age (p-value<0.05), with higher average hospitalization rates in the semiarid region (36.6 per 1,000 live births) and lower rates in Teresina (14.9 per 1,000 live births), as well as an inflection from 2009 with a significant decline in specific macroregions [16]. 

This divergent pattern suggests that local-specific factors are outweighing the benefits of national health policies. A significant territorial inequality among children under 5 years of age was found in 2021 in Arapiraca, with a rate four times higher in rural areas — 26.2 per 1,000 children/year (95%CI 19.4; 34.5) (Table 2). In rural areas of low- and middle-income countries, combined access to improved water and sanitation was associated with a 24% reduction in the occurrence of diarrhea among children under 5 years of age [17]. This reinforces the plausibility that local infrastructure deficits, especially in rural areas, sustain inequities.

In Brazil in 2008, the difference in access to health services between urban and rural areas was 3 percentage points, with half of this difference attributed to unobservable factors, such as a lower supply of nearby services and greater barriers related to travel/distance in rural areas [15]. In this context, structural characteristics of facilities and work processes of primary care teams are associated with the number of hospitalizations for conditions sensitive to primary care in Brazilian municipalities [18], reinforcing the plausibility that access barriers and lower problem-solving capacity within the territory contribute to the persistence of avoidable hospitalizations.

The spatial analysis identified a concentration of hospitalizations in specific areas of the municipality, with three clusters near Lago da Perucaba during the period 2017–2021 (Figure 2). This pattern suggests greater socio-environmental vulnerability in territories with accelerated urbanization and potential exposures associated with sanitation infrastructure and the water environment. In the state of São Paulo, between 2008 and 2012, spatial autocorrelation of hospitalizations for diarrhea in children aged 0 to 4 years was observed (Moran’s I 0.31; p<0.01), with identification of priority municipalities in a high-high pattern, reinforcing the usefulness of the spatial approach to guide targeted interventions [19]. 

The main strength of this study was the integration of temporal and spatial analyses, which allowed the identification of long-term trends and areas of hospitalization concentration, as well as evidence of inequalities between rural and urban areas relevant to health planning. The use of the local SIH/SUS database and manual georeferencing increased spatial accuracy. Therefore, the applicability of the findings to municipal management is reinforced, especially in actions related to sanitation, surveillance of waterborne diseases, and resource allocation.

To improve future analyses, it is recommended to explore monthly series with seasonal decomposition, incorporate environmental and sanitation variables, use spatial models with denominators specific to age groups, and assess readmissions, thereby contributing to monitoring of severity and the care network’s resolution capacity.

## Data Availability

The data analysis files can be accessed at: https://osf.io/u7anf/ [20].
