# Supplementary material for: Hospitalizations for diarrhea in children under 5 years of age: time and spatial series analysis, Arapiraca, 2010–2021
Source: Epidemiol Serv Saude. 2026 Jul 24;35:e20250725. doi: 10.1590/S2237-96222026v35e20250725.en (PMC13686412; doi:10.1590/S2237-96222026v35e20250725.en)
Supplement: Translation

# Translation: Internações por diarreia em crianças menores de 5 anos: análise de série temporal e espacial, Arapiraca, 2010-2021

**DOI:** 10.1590/S2237-96222026v35e20250725.pt

**Authors:** Evandro da Silva Melo, Carla Souza dos Anjos, Lousanny Caires Rocha Melo, Rafaella Souza Albuquerque

**Affiliations:** 1 Universidade Federal Fluminense Faculdade de Medicina Niterói RJ Brasil Universidade Federal Fluminense, Faculdade de Medicina, Niterói, RJ, Brasil; 2 Universidade Federal de Alagoas Programa de Pós-Graduação em Enfermagem Maceió AL Brasil Universidade Federal de Alagoas, Programa de Pós-Graduação em Enfermagem, Maceió, AL, Brasil

**Keywords:** Diarreia, Gastroenterite, Epidemiologia, Estudos de Séries Temporais, Análise Espacial

## Abstract

**Objetivo:**

Analisar a tendência temporal e a distribuição espacial das internações por diarreia em crianças menores de 5 anos em Arapiraca, Alagoas, entre 2010 e 2021.

**Métodos:**

Trata-se de análise de série temporal e espacial das internações por diarreia em menores de 5 anos em Arapiraca. A tendência temporal das taxas de internação foi avaliada por regressão linear autorregressiva entre 2010 e 2019, estimando-se a variação percentual anual média (VPAM), o intervalo de confiança de 95% (IC95%) e o respectivo p-valor. Foi construído um mapa de densidade para avaliar a distribuição espacial utilizando o estimador de densidade de Kernel, para identificar aglomerado de casos entre 2017 e 2021.

**Resultados:**

Foram registradas 4.013 internações; 59,9% ocorreram em crianças menores de 2 anos, 57,1% permaneceram internadas de 2 a 3 dias, e houve 7 óbitos. Em 2021, as taxas de internação em menores de 1 ano foram 4 vezes superiores na zona rural (40,3/1 mil crianças/ano; IC95% 26,8; 64,7). A tendência foi crescente em internações de crianças menores de 5 anos nas zonas rural (VPAM 0,56; IC95% 0,29; 0,83; p-valor 0,001) e urbana (VPAM 0,06; IC95% 0,02; 0,09; p-valor 0,006) e em crianças de 1 a 4 anos, mantendo-se estacionária entre menores de 1 ano. A análise espacial identificou aglomerados de casos em áreas de urbanização acelerada.

**Conclusão:**

A diarreia consiste em um dos principais agravos de internação infantil, com tendências crescentes em territórios vulneráveis, o que reforça a necessidade da vigilância e do planejamento em saúde.

**Aspectos éticos**

Esta pesquisa respeitou os princípios éticos, obtendo os seguintes dados de aprovação:

Comitê de ética em pesquisa Universidade Federal Fluminense

Número do parecer 5.753.066

Data de aprovação 10/11/2022

Certificado de apresentação de apreciação ética 64248922.0.0000.5243

Registro do consentimento livre e esclarecido Dispensado.

## Introdução

Diarreia e gastroenterite de origem infecciosa presumível são doenças infecciosas do trato gastrointestinal caracterizada pelo aumento do número e do volume hídrico das evacuações. Na 10ª revisão da Classificação Internacional de Doenças (CID-10), seu código é o A09 [1].

Em 2021, estimou-se que as doenças diarreicas foram responsáveis por aproximadamente 1,17 milhão de mortes em todo o mundo (intervalo de confiança de 95%, IC95% 0,793; 1,62 milhão), o que representou a redução de 60% em relação a 1990, quando esse número era estimado em 2,93 milhões. A diminuição mais acentuada foi observada em crianças menores de 5 anos, cuja mortalidade por diarreia caiu cerca de 79% [2]. No Distrito Federal (2003-012) e em âmbito nacional (2000-2015), registrou-se que a magnitude das taxas de internação dessa causa é elevada em menores de 1 ano [3,4], com grande impacto em toda população menor de 5 anos [5].

Entre as variáveis relacionadas à diarreia, estão a área geográfica em que a criança reside, o número de pessoas residentes por cômodo, a idade da mãe e da criança [6], a indisponibilidade de rede de esgotos na rua, o acondicionamento irregular de lixo e a presença de vetores intradomiciliares [6,7]. Além de fatores etiológicos de origem viral e bacteriana, acrescentam-se a renda familiar, a frequência à creche/escola [7] e a falta de acesso à água potável [6]. O acesso aos serviços de atenção primária à saúde é considerado fator protetor para a diarreia [7]. A vacinação contra o rotavírus constitui importante fator protetor, associando-se à redução significativa das hospitalizações e dos óbitos por diarreia em crianças menores de 5 anos no Brasil [8].

Em Mato Grosso, entre 2011 e 2020, evidenciou-se a existência de padrões de agrupamento geoespacial nas taxas de internação por diarreia infantil, o que indicou heterogeneidade municipal e sugeriu que fatores locais (sanitários, socioeconômicos e de acesso à saúde) podem modular o risco de hospitalização [9]. Em Arapiraca, entre 2012 e 2021, foram registradas 4.839 internações de crianças menores de 5 anos de idade por causas sensíveis à atenção primária. Destas, 75% ocorreram devido à diarreia [10].

Apesar dos avanços no controle das doenças diarreicas, a compreensão das desigualdades territoriais relacionadas às internações infantis permanece relevante para o planejamento de ações de vigilância e prevenção Este estudo buscou analisar a tendência temporal e a distribuição espacial das internações por diarreia em crianças menores de 5 anos em Arapiraca, Alagoas, entre 2010 e 2021.

## Métodos

### Delineamento

Trata-se de estudo com análise de série temporal e análise espacial das internações por diarreia (CID-10 A09) em crianças menores de 5 anos residentes e internadas em Arapiraca, Alagoas. A análise de tendência temporal considerou o período 2010-2019, a partir de taxas agregadas por faixa etária e zona de residência. A análise espacial incluiu as internações de 2017 a 2021 e utilizou endereços georreferenciados e anonimizados para descrever a distribuição espacial dos eventos. 

### Contexto

A pesquisa foi realizada em Arapiraca, município do agreste de Alagoas. Em 2022, o Instituto Brasileiro de Geografia e Estatística (IBGE) estimou 234.696 habitantes, sendo 7,2% crianças menores de 5 anos. A taxa de mortalidade infantil foi de 14,05 óbitos por 1 mil nascidos vivos em 2021, e 14,19 em 2023. 

Quanto às condições domiciliares, 20,7% dos domicílios contavam com rede de esgoto, 77,6% eram abastecidos pela rede geral de água, e 98,6% dispunham de banheiro de uso exclusivo [11]. Entre 2012 e 2021, 75,0% das internações por condições sensíveis à atenção primária em menores de 5 anos foram atribuídas à diarreia, segundo o Sistema de Informações Hospitalares do Sistema Único de Saúde (SIH/SUS) [10].

### Participantes

Foram incluídas todas as internações registradas no SIH/SUS de crianças menores de 5 anos residentes em Arapiraca, Alagoas, cuja causa básica foi CID-10 A09, considerando-se apenas diagnósticos principais. Essa opção aumenta a especificidade, embora possa subestimar casos classificados como diagnósticos secundários. 

Foram incluídas internações realizadas em unidades públicas, privadas ou filantrópicas prestadoras do SUS no município. Adotou-se o critério de analisar apenas residentes internados no próprio território devido à disponibilidade de variáveis geográficas completas na base municipal do SIH. 

No período analisado, 4.020 internações foram registradas, das quais 4.013 (99,8%) ocorreram em hospitais de Arapiraca. Cada internação foi tratada como evento independente, sem deduplicação de possíveis reinternações. Isso pode superestimar hospitalizações individuais, mas representa o padrão de uso dos serviços. Todos os registros continham informação válida de idade.

### Variáveis, fontes de dados e mensuração

As informações utilizadas nas análises descritiva e de tendência temporal foram obtidas do SIH/SUS, acessadas por meio do portal do Departamento de Informática do SUS (DATASUS) [11].

As variáveis analisadas incluíram:

- unidade hospitalar de internação (Casa de Saúde e Maternidade Nossa Senhora de Fátima, Hospital Regional de Arapiraca, Hospital Chama, Real Hospital Santa Maria);
- faixa etária (0-<1, 1-<2, 2-<3, 3-<4, 4-<5 anos);
- sexo (masculino, feminino);
- raça/cor da pele (branca, preta, parda, amarela, indígena, ignorado);
- condição de alta (alta, permanência por características própria da doença, transferido para outro estabelecimento, óbito, encerramento administrativo); e
- tempo de internação (≤1, 2-3, 4-5, ≥6 dias).

As análises foram estratificadas segundo a zona de residência (rural, urbano, ignorado). A variável raça/cor da pele apresentou ausência de informação em 13,1% dos registros; optou-se por manter a categoria “ignorado” nas tabelas descritivas.

Para a análise espacial, utilizou-se a base local do SIH municipal, que contém informações adicionais não disponíveis no DATASUS, como Código de Endereçamento Postal (CEP) e logradouro. Assim, foi possível geocodificar os casos e elaborar os mapas. Os dados foram extraídos em março de 2023 e analisados de forma anonimizada.

Para o cálculo das taxas, o numerador correspondeu às internações com diagnóstico principal CID-10 A09 registradas no SIH/SUS entre 2010 e 2021. O denominador foi a população anual estimada de crianças menores de 5 anos, estratificada por faixa etária (<1; 1-4; <5) e zona de residência. As taxas foram expressas por 1 mil crianças/ano.

### Análise estatística

O teste qui-quadrado de Pearson foi utilizado para identificar associações entre a zona de residência da criança (rural e urbana) e as variáveis descritivas estudadas, considerando o nível de significância de 5%.

Para estimar o IC95% das taxas de internação, foi utilizado o método estatístico de distribuição de Poisson tratado como um caso especial de distribuição binomial. Este foi utilizado quando o número de eventos a considerar no numerador foi muito pequeno em relação à população referência no denominador. 

As taxas de internação foram tabuladas em planilha do Microsoft Excel após a instalação de um suplemento denominado “conf int” com download no statpages.info. Para calcular o intervalo de confiança inferior, utilizou-se a fórmula PoisLow; para o superior, o argumento PoisHigh.

### Estimativa da população de crianças segundo região rural e urbana

Considerando que o IBGE não disponibiliza estimativas intercensitárias estratificadas por zona de residência, este estudo adotou essa metodologia para interpolar e extrapolar as populações anuais, assumindo crescimento constante entre 2000 e 2021.

A população de crianças estimada para cada ano (2011-2021) foi calculada usando o método geométrico, em que a população cresce proporcionalmente de acordo com uma constante (coeficiente r). As referências de cálculos foram os dados do Censo Demográfico 2000 (P0) e do Censo Demográfico 2010 (Pt) [10]. A Equação 1 foi usada para obter o coeficiente r:

Equação 1: $r=[Pt/P0{]}^{\left[1/(t-t0)\right]}-1$

em que t correspondeu ao número de anos contados a partir de 1º de agosto de 2010 até 1º de agosto de 2021. Já a Equação 2 serviu para obter a população projetada: 

Equação 2: $Pt=P0[(1+r{)}^{(t),}]$

em que t é o marco temporal que necessita ser projetado. 

As estimativas foram obtidas para a população de crianças, desagregada por faixa etária (<1, 1-4, <5) e por região de residência (rural, urbana). Os valores projetados foram comparados às estimativas intercensitárias oficiais do IBGE e apresentaram diferença média de aproximadamente 5%. Essa discrepância refletiu pequenas flutuações demográficas locais não captadas pelo crescimento médio intercensitário, mas não comprometeu a coerência das tendências temporais, uma vez que o mesmo método foi aplicado uniformemente a todo o período analisado.

### Análise da tendência temporal

Esta etapa se iniciou pela inspeção de gráficos de linha, com as taxas observadas plotadas no eixo y e os anos da série plotados no eixo x. A análise das tendências temporais das taxas de internação e de regressão linear autorregressiva foi empregada com o método estatístico dos mínimos quadrados ordinários e os pacotes “car” e “nlme” disponibilizados na plataforma R. O método foi adequado para lidar com a falta de independência dos dados (anos da série) e permitiu ajustar modelos considerando defasagens temporais e ajustando para “n lags” conforme avaliado pela função de autocorrelação serial. 

As taxas de internação por diarreia foram transformadas em logaritmo de base 10 (log₁₀) com o objetivo de estabilizar a variância e atender aos pressupostos de normalidade. Foram considerados dois termos de defasagem (AR(2)).

A magnitude e a direção da tendência foram expressas pela variação percentual anual média (VPAM), calculada a partir do coeficiente angular (β_1_) do modelo. Este foi obtido de acordo com o percurso metodológico adotado por Antunes e Cardoso [12], segundo a expressão:


$$VPAM=(10^{\beta_{1}}-1)\times 100$$

Os limites inferior e superior do IC95% foram obtidos, respectivamente, por:


$$LI=(10^{\beta_{1min}}-1)\times 100eLS=(10^{\beta_{1max}}-1)\times 100$$

As tendências foram classificadas como crescentes quando o IC95% foi inteiramente positivo, decrescentes quando inteiramente negativo e estacionárias quando resultavam em zero. As tendências temporais foram analisadas usando o software R version 4.1.0 (2021-05-18), utilizando-se os dados tabulados em planilhas do Excel 2016. Para a análise de tendência temporal, foi considerado o período 2010-2019.

### Distribuição espacial

A distribuição espacial incluiu internações por diarreia em crianças menores de 5 anos no período 2017-2021, utilizando o SIH/SUS local, que disponibiliza CEP e logradouro de residência. Os endereços foram georreferenciados manualmente no software Google Earth Pro (versão 7.3; Google LLC), gerando coordenadas validadas caso a caso. A taxa de sucesso foi de 90,9%; endereços incompletos ou ambíguos foram padronizados ou representados pelo centroide do logradouro/bairro.

As coordenadas foram convertidas em camada de pontos no QGIS 3.16 e sobrepostas à malha censitária municipal disponibilizada pelo IBGE. A densidade espacial foi estimada pelo método de Kernel, adotando largura de banda de 500 m e sistema de projeção Datum SIRGAS 2000/UTM 24S. Trata-se de parâmetros selecionados conforme a escala territorial do município para identificar áreas de maior concentração de internações.

## Resultados

Entre 2010 e 2021, ocorreram 4.013 internações por diarreia em crianças menores de 5 anos em Arapiraca, registradas exclusivamente em unidades vinculadas ao SUS. Uma única instituição hospitalar foi responsável pela notificação de 60,18% (n=2.415) das internações (Tabela 1).

**Tabela 1:** Distribuição quantitativa das internações por diarreia de crianças menores de 5 anos de idade residentes e internadas no âmbito do Sistema Único de Saúde, por unidade hospitalar de internação, sexo, faixa etária (meses), raça/cor da pele, evolução, tempo de internação (dias) e zona de residência. Arapiraca, 2010-2021 (n=4.013)

Variáveis			Zona de residência		
		Total n	Ignorado n (%)	Rural n (%)	Urbana n (%)
**Unidade hospitalar de internação**					
Casa de Saúde e Maternidade Nossa Senhora de Fátima		2.415	34 (51,0)	743 (64,0)	1.638 (59,0)
Hospital Regional de Arapiraca		351	1 (1,5)	81 (7,0)	269 (9,6)
Hospital Chama		867	5 (7,5)	220 (19,0)	642 (23,0)
Real Hospital Santa Maria		380	27 (40,0)	110 (9,5)	243 (8,7)
Sexo					
Feminino		1.943	26 (39,0)	550 (48,0)	1.367 (49,0)
Masculino		2.070	41 (61,0)	604 (52,0)	1.425 (51,0)
**Faixa etária **(anos)					
0-<1		1.221	28 (42,0)	367 (32,0)	826 (30,0)
1-<2		1.186	18 (27,0)	351 (30,0)	817 (29,0)
2-<3		755	12 (18,0)	205 (18,0)	538 (19,0)
3-<4		516	4 (6,0)	133 (12,0)	379 (14,0)
4-<5		335	5 (7,5)	98 (8,5)	232 (8,3)
**Raça/cor da pele**					
Branca		67	3 (4,5)	20 (1,7)	44 (1,6)
Preta		2	-	-	2 (0,1)
Parda		3.418	33 (49,3)	992 (86,0)	2.393 (85,7)
Amarela		-	-	-	-
Indígena		-	-	-	-
Ignorada		526	31 (46,3)	142 (12,3)	353 (12,6)
Evolução					
Alta		3.979	67	1.141	2.771
Permanência por características própria da doença		2	-	1 (<0,1)	1 (<0,1)
Transferido para outro estabelecimento		24	-	7 (0,6)	17 (0,6)
Óbito		7	-	5 (0,4)	2 (<0,1)
Encerramento administrativo		1	-	-	1 (<0,1)
**Tempo de internação **(dias)					
≤1		16	-	5(0,4)	11(0,4)
2-3		2.293	47(70,0)	691(59,9)	1.555(55,6)
4-5		1.222	15(22,0)	332(28,7)	875(31,0)
≥6		482	5(7,5)	126(10,9)	351(13,0)

As hospitalizações foram mais frequentes em menores de 2 anos de idade (59,9%), sem diferença significativa entre os sexos (p-valor 0,457), e o tempo de internação mostrou-se menor em crianças da zona rural. A variável raça/cor da pele apresentou predominância de crianças pardas (85,7%), seguida por registros sem informação (13,1%). A incompletude da informação foi mais elevada entre residentes da zona rural (46,3%), o que evidenciou desigualdade na qualidade do registro. Não houve diferença estatisticamente significativa na distribuição de raça/cor da pele entre as zonas de residência (p-valor 0,733) (Tabela 1).

As taxas de internação apresentaram valores mais elevados na zona rural durante todo o período, especialmente entre menores de 1 ano, que concentraram as maiores incidências. Em 2021, as taxas na zona rural foram 4 vezes superiores às da zona urbana (40,3 versus 9,9 por 1 mil crianças/ano). Entre menores de 1 ano, as taxas foram quase o dobro das observadas entre crianças de 1 a 4 anos, tanto na zona urbana (9,9 vs. 5,0 por 1 mil crianças/ano) quanto na zona rural (40,3 vs. 21,8 por 1 mil crianças/ano) (Tabela 2).

**Tabela 2:** Taxas de internação (TI) por diarreia (por 1 mil crianças/ano) de menores de 5 anos de idade e respectivos intervalos de confiança de 95% (IC95%), no âmbito do Sistema Único de Saúde, por faixa etária estratificada, ano de internação e zona de residência. Arapiraca, 2010-2021 (n=4.013)

Faixa etária (anos)	Ano de internação	TI zona rural (IC95%)	TI zona urbana (IC95%)	TI total (IC95%)
<1	2010	82,0 (60,8; 108,1)	32,1 (25,9; 39,2)	44,1 (37,4; 51,5)
	2011	48,9 (32,8; 70,2)	18,3 (13,7; 24,0)	24,1 (19,2; 29,8)
	2012	46,8 (30,8; 68,1)	21,0 (16,0; 27,1)	26,2 (21,1; 32,3)
	2013	78,6 (57,1; 105,5)	39,8 (32,7; 47,8)	46,8 (39,8; 54,7)
	2014	66,1 (46,3; 91,4)	26,6 (20,9; 33,4)	33,4 (27,5; 40,2)
	2015	54,7 (36,6; 78,6)	22,2 (17,0; 28,6)	28,8 (23,2; 35,2)
	2016	42,7 (26,8; 64,7)	25,8 (20,2; 32,7)	28,9 (23,3; 35,4)
	2017	83,8 (60,4; 113,3)	35,2 (28,4; 43,1)	42,9 (36,0; 50,8)
	2018	73,9 (51,8; 102,3)	28,4 (22,4; 35,7)	36,3 (29,9; 43,6)
	2019	65,5 (44,5; 93,0)	24,2 (18,6; 31,0)	31,0 (25,1; 37,9)
	2020	6,5 (1,3; 19,1)	17,5 (12,8; 23,4)	15,9 (11,7; 21,1)
	2021	40,3 (23,9; 63,6)	9,9 (6,4; 14,6)	14,5 (10,5; 19,5)
1-4	2010	39,6 (31,9; 48,6)	19,0 (16,6; 21,6)	23,1 (20,6; 25,7)
	2011	19,9 (14,5; 26,8)	10,3 (8,6; 12,3)	12,2 (10,4; 14,2)
	2012	26,9 (20,4; 34,9)	14,6 (12,5; 16,9)	16,6 (14,6; 18,9)
	2013	45,7 (36,9; 56,0)	21,1 (18,5; 23,9)	24,9 (22,3; 27,8)
	2014	38,4 (30,2; 48,1)	16,7 (14,4; 19,2)	20,1 (17,7; 22,6)
	2015	22,9 (16,6; 30,9)	10,8 (9,0; 12,9)	12,9 (11,0; 15,0)
	2016	35,5 (27,4; 45,4)	17,5 (15,1; 20,2)	20,5 (18,1; 23,2)
	2017	68,2 (56,5; 81,7)	20,5 (17,9; 23,4)	27,5 (24,7; 30,5)
	2018	42,8 (33,4; 53,9)	16,2 (13,9; 18,7)	20,0 (17,6; 22,7)
	2019	39,5 (30,4; 50,6)	14,9 (12,7; 17,5)	18,5 (16,1; 21,1)
	2020	23,5 (16,5; 32,6)	8,8 (7,1; 10,8)	10,9 (9,1; 12,9)
	2021	21,8 (14,9; 30,7)	5,0 (3,7; 6,5)	7,2 (5,8; 8,9)
<5	2010	48,5 (40,8; 57,2)	21,5 (19,2; 24,0)	27,3 (24,9; 29,8)
	2011	26,1 (20,5; 32,8)	11,9 (10,2; 13,8)	14,6 (12,8; 16,5)
	2012	31,2 (24,9; 38,6)	15,8 (13,9; 18,0)	18,6 (16,6; 20,7)
	2013	52,8 (44,4; 62,5)	24,8 (22,3; 27,5)	29,3 (26,8; 32,0)
	2014	44,5 (36,6; 53,6)	18,6 (16,4; 21,0)	22,8 (20,5; 25,2)
	2015	30,0 (23,4; 37,7)	13,0 (11,2; 15,1)	16,1 (14,2; 18,1)
	2016	37,2 (29,7; 45,9)	19,1 (16,9; 21,6)	22,2 (20,0; 24,6)
	2017	71,8 (61,1; 83,9)	23,4 (20,9; 26,1)	30,6 (27,9; 33,4)
	2018	49,9 (40,9; 60,3)	18,6 (16,3; 21,0)	23,3 (20,9; 25,8)
	2019	45,6 (36,8; 55,8)	16,8 (14,6; 19,1)	21,0 (18,8; 23,4)
	2020	19,6 (14,0; 26,8)	10,5 (8,8; 12,4)	11,9 (10,2; 13,8)
	2021	26,2 (19,4; 34,5)	5,9 (4,7; 7,4)	8,7 (7,2; 10,3)

O comportamento temporal foi cíclico, com picos em 2013 e 2017, redução em 2020 e aumento em 2021. As taxas da zona rural mantiveram-se consistentemente superiores às da urbana, sem sobreposição dos intervalos de confiança (Figura 1). As análises entre 2010 e 2019 revelaram tendência crescente nas taxas de internação por diarreia entre crianças menores de 5 anos, tanto na zona rural (VPAM 0,56; IC95% 0,29; 0,83) quanto na urbana (VPAM 0,06; IC95% 0,02; 0,09). Entre crianças de 1 a 4 anos, observaram-se padrões semelhantes na zona rural (VPAM 0,62; IC95% 0,38; 0,87) e na urbana (VPAM 0,05; IC95% 0,01; 0,09). Para menores de 1 ano, a tendência manteve-se estacionária na zona rural (VPAM 0,19; IC95% -0,59; 0,99) e crescente na zona urbana (VPAM 0,06; IC95% 0,01; 0,^11^). Considerando o conjunto total de crianças menores de 5 anos, verificou-se tendência geral de aumento das internações (VPAM 0,11; IC95% 0,05; 0,^17^) (Tabela 3).

**Tabela 3:** Tendência temporal das taxas de internação por diarreia (por 1 mil crianças/ano) de menores de 5 anos de idade e respectivas variações percentuais anuais médias (VPAM), intervalos de confiança de 95% (IC95%) e p-valor, por zona de residência e faixa etária. Arapiraca, 2010-2019 (n=3.714)

Zona de residência	Faixa etária (anos)	Tendência temporal	VPAM (IC95%)	p-valor
Rural	<5	Crescente	0,56 (0,29; 0,83)	0,001
	<1	Estacionária	0,19 (-0,59; 0,99)	0,596
	1-4	Crescente	0,62 (0,38; 0,87)	<0,001
Urbana	<5	Crescente	0,06 (0,02; 0,09)	0,006
	<1	Crescente	0,06 (0,01; 0,11)	0,030
	1-4	Crescente	0,05 (0,01; 0,09)	0,014
Total	<5	Crescente	0,11 (0,05; 0,17)	0,004
	<1	Estacionária	0,07 (-0,09; 0,23)	0,358
	1-4	Crescente	0,12 (0,07; 0,17)	<0,001

**Figura 1:** Representação gráfica das taxas de internação por diarreia (por 1 mil crianças/ano) e respectivos intervalos de confiança de 95% (IC95%) de menores de 5 anos de idade, segundo ano de internação e zona de residência. Arapiraca, 2010-2021 (n=4.013)

A análise espacial do conjunto de internações ocorridas entre 2017 e 2021 evidenciou quatro aglomerados de maior densidade, concentrados principalmente nas regiões central e sudeste do município. As áreas de maior densidade (≥28,5 casos/km^2^, raio 500 m) localizaram-se nos bairros Manoel Teles, Primavera e Olho D’Água dos Cazuzinhas, próximos ao Lago da Perucaba, e um quarto aglomerado foi identificado no Residencial Agreste, ao norte. Os três primeiros aglomerados estavam em áreas cobertas por unidades básicas de saúde, enquanto o quarto encontrava-se distante dos serviços. Tal resultado indicou descompasso espacial entre a ocorrência das internações e a cobertura assistencial. Isso evidenciou distribuição espacial não coincidente entre a ocorrência das internações e a localização das unidades básicas de saúde (Figura 2).

**Figura 2:** Distribuição espacial das internações por diarreia em crianças menores de 5 anos de idade. Arapiraca, 2017-2021 (n=1.335)

## Discussão

As internações por diarreia em crianças menores de 5 anos em Arapiraca mantiveram-se elevadas ao longo da série histórica, com predomínio entre menores de 2 anos e tendência crescente das taxas, sobretudo na zona rural. A identificação de aglomerados de casos em áreas periféricas e de urbanização recente reforça a influência do contexto ambiental e da infraestrutura domiciliar sobre a ocorrência do agravo. Esses achados indicam que a diarreia continua sendo importante causa de hospitalização infantil no município, o que reflete desigualdades territoriais e socioambientais que influenciam o risco de adoecimento.

Este estudo apresentou limitações inerentes ao uso de dados secundários provenientes do SIH/SUS, passíveis de subnotificação, erros de codificação e inconsistências no preenchimento de variáveis, especialmente endereço e raça/cor da pele. A possível imprecisão na codificação diagnóstica do CID-10 A09, que agrega diferentes causas de diarreia, pode gerar classificação incorreta dos casos e afetar a precisão das taxas de internação. Além disso, o SIH/SUS contempla apenas internações financiadas pelo SUS, o que pode subestimar a ocorrência do agravo em unidades exclusivamente privadas. 

As estimativas populacionais utilizadas no cálculo das taxas, baseadas em projeções intercensitárias do IBGE (Censos Demográficos 2000 e 2010), também podem introduzir margens de erro. Isso se dá sobretudo para os anos de 2020 e 2021, que extrapolam o intervalo censitário, apesar da diferença média de 5% em relação às estimativas oficiais recentes, considerada aceitável para análises municipais.

Na análise espacial, o uso do estimador de densidade Kernel em pontos individuais permitiu identificar aglomerações espaciais de internações, mas não ajustou as densidades pela população em risco. As concentrações podem refletir maior densidade populacional infantil e/ou condições socioambientais de vulnerabilidade. Os resultados também podem ter sido influenciados pelos parâmetros de suavização (largura de banda e tamanho de célula) e por erros de geocodificação, decorrentes da ausência ou inconsistência de CEP em parte dos registros. 

A elevada proporção de internações em menores de 2 anos (60%), sem diferença significativa entre os sexos, está alinhada com padrões observados em outros estudos brasileiros. Na Ilha de Guaratiba, Rio de Janeiro, em 2016, identificou-se padrão etário semelhante, em que crianças menores de 2,5 anos apresentavam maior risco de diarreia [7]. Taxas mais elevadas de hospitalização por diarreia em crianças menores de 1 ano foram registradas no Distrito Federal (27%) em 2012 [3]. Apesar de essas taxas serem muito inferiores às observadas em Arapiraca, esse achado é particularmente relevante considerando que a diarreia aguda constitui uma das principais causas de morbimortalidade infantil no Brasil, com impacto significativo na qualidade de vida e no desenvolvimento infantil. 

Em 2021, demonstrou-se que, embora a carga global das doenças diarreicas tenha diminuído nas últimas décadas no mundo todo (redução de 60% das mortes entre 1990 e 2021), ainda persistem desigualdades territoriais expressivas, concentradas em regiões e grupos populacionais com piores condições de infraestrutura e menor cobertura de serviços de saúde [2]. No município de São Paulo, a vigilância epidemiológica das doenças diarreicas permitiu identificar o norovírus como principal agente etiológico entre os casos com confirmação laboratorial no período analisado. Entre 2010 e 2016, foram registradas 1.565 notificações que atendiam à definição de caso, das quais 444 (28%) foram confirmadas para norovírus e 368 (23%) para rotavírus. Esses achados reforçam a importância da vigilância das doenças diarreicas produzindo informações que ampliam a compreensão da dinâmica desses agravos na infância [13].

Em Arapiraca, a influência dos determinantes ambientais torna-se evidente ao comparar os coeficientes de internação entre as populações rural e urbana. O acesso aos serviços de saúde figura entre os principais determinantes da qualidade de vida e do desenvolvimento socioeconômico, sendo uma das dimensões utilizadas pelo Banco Mundial para a mensuração multidimensional da pobreza [14]. Mesmo diante de condições socioeconômicas semelhantes, a acessibilidade aos serviços de saúde tende a ser mais restrita nas regiões rurais em comparação às áreas urbanas [15]. Em Arapiraca, em 2022, cerca de 92% da população residia em área urbana; apenas 21% dos domicílios estavam conectados à rede de esgoto, e 78% contavam com abastecimento de água pela rede geral [11]. 

É notável que este estudo tenha identificado tendência crescente nas taxas de internação por diarreia em crianças menores de 5 anos em Arapiraca, especialmente na zona rural, contrastando com padrões de redução descritos em outros contextos brasileiros. Enquanto em Mato Grosso as taxas anuais de hospitalização por diarreia em menores de 1 ano diminuíram de 8,50 para 3,45/1 mil nascidos vivos entre 2011 e 2020 [9], em Arapiraca a tendência manteve-se estacionária para esse mesmo grupo etário (VPAM 0,07; IC95% -0,09; 0,23; p-valor 0,358). No Piauí, no período 2000-2019, também se observou tendência de diminuição das internações e da mortalidade por doenças diarreicas agudas em menores de 5 anos (p-valor<0,05), com maiores taxas médias de internação no semiárido (36,6/1 mil nascidos vivos) e menores em Teresina (14,9/1 mil nascidos vivos), além de inflexão a partir de 2009 com queda significativa em macrorregiões específicas [16]. 

Esse padrão divergente sugere que fatores locais específicos estão superando os benefícios das políticas nacionais de saúde. Importante desigualdade territorial entre os menores de 5 anos foi encontrada em 2021, em Arapiraca, com taxa quatro vezes superior na zona rural - 26,2 por 1 mil crianças/ano (IC95% 19,4; 34,^5^) (Tabela 2). Em áreas rurais de países de baixa e média renda, o acesso conjunto a água e saneamento melhorados associou-se à redução de 24% na ocorrência de diarreia em menores de 5 anos [17]. Isso reforçou a plausibilidade de que déficits locais de infraestrutura, sobretudo em áreas rurais, sustentem iniquidades.

No Brasil, em 2008, a diferença no acesso a serviços de saúde entre áreas urbanas e rurais foi de 3 pontos percentuais, sendo metade atribuída a fatores não observáveis, como menor oferta de serviços nas proximidades e maiores barreiras de deslocamento/distância nas áreas rurais [15]. Nesse contexto, características de estrutura das unidades e de processo de trabalho das equipes na atenção primária associam-se ao número de internações por condições sensíveis à atenção primária em municípios brasileiros [18], reforçando a plausibilidade de que barreiras de acesso e menor resolutividade no território contribuam para a persistência das hospitalizações evitáveis.

A análise espacial identificou concentração de internações em áreas específicas do município, com três aglomerados próximos ao Lago da Perucaba, no período 2017-2021 (Figura 2). Esse padrão sugere maior vulnerabilidade socioambiental em territórios com urbanização acelerada e possíveis exposições relacionadas à infraestrutura sanitária e ao ambiente hídrico. No estado de São Paulo, entre 2008 e 2012, observou-se autocorrelação espacial das internações por diarreia em crianças de 0 a 4 anos (I de Moran 0,31; p-valor<0,01), com identificação de municípios prioritários em padrão alto-alto, reforçando a utilidade da abordagem espacial para orientar intervenções focalizadas [19]. 

A principal força deste estudo foi a integração das análises temporal e espacial, o que permitiu identificar tendências de longo prazo e áreas de concentração das internações, além de evidenciar desigualdades entre zonas rural e urbana relevantes para o planejamento em saúde. O uso da base local do SIH/SUS e o georreferenciamento manual aumentaram a precisão espacial. Reforça-se, portanto, a aplicabilidade dos achados para a gestão municipal, especialmente em ações de saneamento, vigilância de doenças de veiculação hídrica e alocação de recursos.

Para aprimorar futuras análises, recomendam-se explorar séries mensais com decomposição sazonal, incorporar variáveis ambientais e de saneamento, utilizar modelos espaciais com denominadores específicos por faixa etária e avaliar reinternações, contribuindo para o monitoramento da gravidade e da resolutividade da rede assistencial.

## Data Availability

Os arquivos com análise dos dados podem ser consultados em: https://osf.io/u7anf/ [20].
